# Hepatotoxicity Associated with Use of the Weight Loss Supplement* Garcinia cambogia*: A Case Report and Review of the Literature

**DOI:** 10.1155/2018/6483605

**Published:** 2018-03-12

**Authors:** Jiten P. Kothadia, Monica Kaminski, Hrishikesh Samant, Marco Olivera-Martinez

**Affiliations:** ^1^Nazih Zuhdi Transplant Institute, INTEGRIS Baptist Medical Center, 3300 NW Expressway, Oklahoma City, OK 73112, USA; ^2^Department of Internal Medicine, Coney Island Hospital, 2601 Coney Island Avenue, Brooklyn, NY 11235, USA; ^3^Division of Gastroenterology and Hepatology, Louisiana State University Health Sciences Center, Shreveport, LA 71103, USA; ^4^Department of Gastroenterology and Hepatology, University of Nebraska Medical Center, 982000 Nebraska Medical Center, Omaha, NE 68198, USA

## Abstract

The use of herbal and dietary supplements for weight loss is becoming increasingly common as obesity is becoming major health problem in the United States. Despite the popularity of these natural supplements, there are no guidelines for their therapeutic doses and their safety is always a concern.* Garcinia cambogia* extract with its active ingredient “hydroxycitric acid” is a component of many weight loss regimens. It suppresses fatty acid biosynthesis and decreases appetite. However, its prolonged use in weight maintenance is unknown. Here we describe a case of acute hepatitis after the use of* Garcinia cambogia* for weight loss.

## 1. Introduction

In the United States, dietary supplements (DS) are being used more commonly as a strategy for weight loss [1–3]. The National Health and Nutrition Examination Survey (NHANES) 2003–2006 showed the use of DS in as many as 50% of Americans and 70% of adults above the age of 70 years [3, 4]. Many consumers have a false sense of security that these products are “natural” and thus safe for use [2, 3, 5]. In reality, many of these DS do not have established guidelines for safe doses and their use is not as tightly regulated by the United States (US) Food and Drug Administration (FDA) as pharmaceuticals [1, 3, 5]. For pharmaceuticals to be approved for the market, there is a process of close scientific scrutiny including a demonstration of safety and efficacy; no such scrutiny is applied to dietary supplements as they are considered as food products [1, 4]. Some DS also have been associated with significant side effects and case reports of DS causing such health injuries are increasing [2, 5]. It is often challenging to determine the causative agent, as many of these DS are made up of a variety of compounds that may change with time [3]. Although direct causality is difficult to confirm, the US Drug Induced Liver Injury Network (DILIN) has reported that herbal supplements attributed liver injury has increased in the past ten years and ranges from 2% to 16% of all reported hepatotoxicity [3, 4]. In particular, DS known to cause liver injury include Hydroxycut,* Camellia sinensis* (green tea extract), Herbalife products, usnic acid, LipoKinetix, 1,3-Dimethylamylamine, uncoupling protein-1, vitamin A, OxyELITE pro, and anabolic steroids [4].

*Garcinia cambogia (GC)* is a component of many dietary supplements for weight loss.* GC* is a tropical fruit that grows in South East Asia and Africa and has been found to contain hydroxycitric acid (HCA) in its rind [3, 6, 7]. This active ingredient is an inhibitor of adenosine triphosphate (ATP) citrate lyase, which is an enzyme involved in fatty acid biosynthesis and glycogen storage. It also causes suppression of appetite [2, 8]. Thus,* GC*/HCA is often added to weight loss products [3, 4, 7]. Its potential to cause health hazard remains controversial, but there has been evidence in animal studies to show that* GC* is linked to causing oxidative stress, inflammation, and hepatic fibrosis [3, 4, 7].

## 2. Case Discussion

A 36-year-old female with no significant medical history presented with a 3-day history of low-grade fever, nausea, vomiting, and abdominal pain. She reported that she had been following a 500 Kcal diet and was taking GC for four weeks to lose weight. She also complained of fatigue, anorexia, and jaundice. The patient denied any history of recent blood transfusion, illicit drug use, or family history of liver disease. She denied alcohol consumption and was in a monogamous heterosexual relationship.

On physical examination, she had scleral icterus, cutaneous jaundice, and tender hepatomegaly measuring 2 cm below the costal margin. Abnormal laboratory results included white blood cell count of 2.73 × 10^3^ cells/*μ*L (4–11) and platelet count of 78 × 10^3^ cells/*μ*L (150–400), aspartate aminotransferase (AST) of 5340 U/L (15–41), alanine aminotransferase (ALT) of 5615 U/L (7–52), alkaline phosphatase of 104 U/L (32–91), total bilirubin of 7.4 mg/dl (0.3–1.0), and direct bilirubin of 4.9 mg/dl (0.0–0.4). Serologies for hepatitis A, hepatitis B, hepatitis C, autoimmune markers (anti-nuclear antibody, anti-smooth muscle antibodies, anti-mitochondrial antibody, and anti-liver kidney microsomal 1 antibody), human immunodeficiency virus, rapid plasma reagin test, cytomegalovirus, Epstein-Barr virus, Herpes Simplex virus, and Parvovirus were negative. Serum ceruloplasmin, alpha-fetoprotein, and alpha-1 antitrypsin levels were normal. Abdominal Doppler ultrasound showed mild echotexture coarsening in the liver and small ascites.

**Figure 1:**
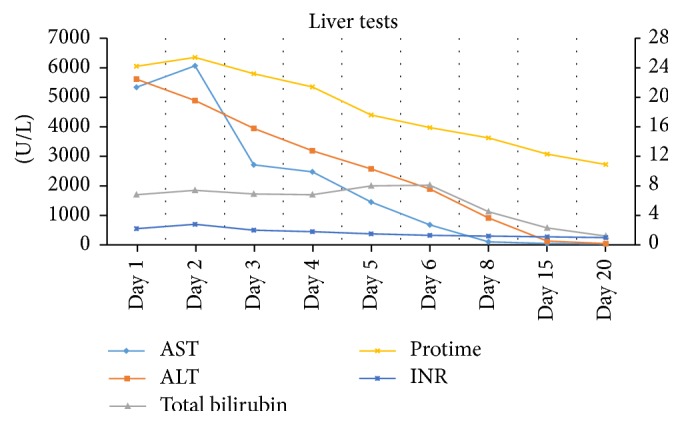
Changes in liver tests during hospitalization are plotted from admission at day 1 to day 20.

Considering her recent exposure to herbal medications, we suspected drug induced liver injury and used updated RUCAM (Roussel Uclaf Causality Assessment Method) scale to calculate its probability. Patient's RUCAM score came out to be 8 points, which is consistent with probable drug induced liver injury [9]. GC was discontinued and conservative management was initiated. Significant clinical improvement and the downward trend of liver function tests obviated the need for liver biopsy. The patient was discharged on hospital day 6. At follow-up visit two weeks later, her liver function tests had returned to normal (Figure 1).

## 3. Discussion

The use of dietary supplements with a perception that such use is safe is becoming increasingly popular in USA [2, 16]. Also, with the epidemic of obesity in US, many people have considered DS as a treatment remedy for weight loss [20]. However, there are no guidelines for their use and the FDA does not tightly regulate these DS for their safety. Several DS have been associated with acute liver injury, including fulminant liver failure requiring liver transplantation [2, 16].

According to the US Congress, DS is defined as a product taken by mouth which contains dietary ingredients to supplement the regular diet. These dietary ingredients include vitamins, herbs, minerals, amino acids, enzymes, metabolites, extracts, or concentrates [16]. Under the Dietary Supplement Health and Education Act (DSHEA), the FDA has the responsibility of demonstrating that a DS is harmful before it can take action to restrict or remove it from the market. In contrast, the pharmaceutical company must prove the safety of the medication it is manufacturing by clinical trials before the FDA will grant their approval [16].

In 2009, the FDA issued a notice against a popular supplement,* Hydroxycut*, due to the associated liver injury and one reported death [2, 3].* GC* is one of the many ingredients contained in this compound, but it is not clear which ingredient of* Hydroxycut* compound was responsible for the liver damage [1, 21]. Furthermore, only 8 of the 14 marketed* Hydroxycut* products contained hydroxycitric acid (HCA) which is also the active component of* GC* [21]. Despite this information, many* Hydroxycut* products are still available online. In our patient, Omnitrition International, INC manufactured the GC supplement and contained GC extract 1000 mg (standardized to 50% HCA) and Potassium 150 mg per serving (2 capsules).

GC is a tropical fruit that grows in Southeastern Asia and Western Africa and contains active ingredient HCA [1, 2, 22]. Studies in both experimental mice and humans have shown fat loss and decrease in body weight [1]. Fat loss and weight reduction occur through many mechanisms including prevention of the conversion of carbohydrates to fatty acids by the inhibition of fatty acid biosynthesis through block of the ATP citrate lyase enzyme, which in turn leads to increased hepatic glycogen synthesis, and finally suppression appetite leading to decreased food intake [3, 21]. Appetite is suppressed further by the increased release of serotonin which is a neurotransmitter associated with eating behavior [21]. HCA has been on sale for almost two decades and there appear to be no reports of human liver toxicity other than those mentioned above regarding the product* Hydroxycut* of which HCA is a component [1, 6, 22].

Kim et al. studied the use of GC in a population of C57BL/6J mice fed a high-fat diet (45 kcal% fat) [1, 21, 22]. After a prolonged duration of observation over 16 weeks, it was determined that the use of* GC* promoted fatty acid oxidation and decreased fatty acid synthesis, leading to the amelioration of adipogenesis [1, 7]. They also showed that it induced oxidative stress, inflammation, and hepatic fibrosis as well as hepatic collagen accumulation and lipid peroxidation [1, 22]. Contrary to other published studies performed on animals and humans, this study by Clouatre and Preuss found HCA of GC to have a protective effect on the liver [22]. Thus, the form of HCA regarding strength, extraction process, residual compounds, and so forth may create a difference in study outcomes and requires definition [22].

Although there have been studies that show the weight loss benefit of* GC*, randomized, double-blind, placebo-controlled trial by Heymsfield et al. showed no significant change in fat mass and body weight observed over those using a placebo at 12 weeks [23]. A recent systematic review and meta-analysis by Onakpoya et al. showed that GC extract could cause short-term weight loss, but its overall effect on long-term weight is uncertain [24].

While it is difficult to prove causation in any drug-induced liver injury (DILI), in our case, hepatotoxicity was seen after taking GC and significant improvement in the liver function tests was seen after its discontinuation. Also, the absence of any other etiologies including infectious, autoimmune, and metabolic causes proven by comprehensive testing was suggestive of the fact that GC was the probable cause of the hepatotoxicity. Our patient was not tested for hepatitis E as hepatitis E is rare in the United States as a cause of acute liver failure. Also, clinical history was not classical for hepatitis E. We have summarized similar cases of hepatotoxicity secondary to GC reported till now in literature in Table 1 [2, 7, 10–20]. All of these cases presented with nonspecific symptoms such as nausea, vomiting, malaise, abdominal pain, and jaundice. The pattern of liver injury was hepatocellular in the majority of cases except for 3 cases that presented with cholestatic pattern. Six patients (24%) required orthotopic liver transplant. These cases indicate the need for better postmarketing surveillance and highlight the importance of reporting such cases to assist this process further.

DS induced liver injury often continues to remain a diagnosis of exclusion once viral hepatitis, autoimmune causes, and metabolic disturbances are excluded [2, 3]. Thus it is important to keep in mind that there may be further workup required to diagnose a DILI [2]. Despite this caveat, it is of benefit to obtain a thorough history of herbal or dietary supplements when the etiology of liver injury is unclear, both for the benefit of choosing appropriate therapy for the patient and for the future of drug development [2].

## 4. Conclusion

Although DS are often perceived to be natural and safe, they frequently have harmful side effects and can result in significant morbidity and mortality. This case depicts hepatotoxicity that was associated with the use of weight loss supplement* GC*. The physician should always ask about the use of DS as many patients may fail to disclose this information. Our case indicates the need for better postmarket surveillance and highlights the importance of reporting such cases to assist this process further.

## Figures and Tables

**Table 1:** Summary of patients with *Garcinia cambogia* related liver injury, characteristics, presentation, pattern of liver injury, and clinical outcome.

Author (year) [ref.]	Number Of cases	Age	Sex	Duration of GC containing supplement use	Presenting symptoms	Pattern of liver injury	Updated RUCAM score	Number of cases that underwent liver transplant	Mortality
Stevens et al. [10] (2005)	2	28.5^*∗*^	M	5 weeks; 5 days	Fatigue, jaundice	Hepatocellular; cholestatic	8^*¥*^	0	0

Jones and Andrews [11] (2007)	1	19	M	120 days	Nausea, vomiting, and jaundice	Hepatocellular	7	0	0

Laczek and Duncan [12] (2008)	3	24.33^*∗*^	M	60-90 days	Malaise, jaundice, and pruritus	2 hepatocellular; 1 cholestatic	8^*¥*^	0	0

Dara et al. [13] (2008)	2	36.5^*∗*^	F	7 days; 14 days	Nausea, vomiting, fatigue, anorexia, and abdominal pain	Hepatocellular	8^*¥*^	0	0

Shim and Saab [14] (2009)	1	28	M	90 days	Fatigue, jaundice	Hepatocellular	8	0	0

Fong et al. [15] (2010)	8	30.9^*∗*^	6M; 2 F	7 to 56 days	Nausea, vomiting, fatigue, and itching abdominal pain	Hepatocellular	7-8^*¥*^ NA for 3 patients needing transplant	3	0

Danan and Teschke [9] (2010)	1	19	M	7 days	Fever, fatigue, myalgia, arthralgia, and rash	Cholestatic	7	0	0

Sharma et al. [16] (2014)	1	27	M	Unknown	Nausea, vomiting, abdominal pain, and jaundice	Hepatocellular	7	0	0

Lee et al. [17] (2014)	1	39	F	2 days	Abdominal pain, anorexia, nausea, dyspepsia, fatigue, and jaundice	Hepatocellular	8	0	0

Melendez-Rosado et al. [7] (2015)	1	42	F	7 days	Nausea, abdominal pain	Hepatocellular	7	0	0

Corey et al. [2] (2016)	1	52	F	25 days	Jaundice, decreased appetite, fatigue, and confusion	Hepatocellular	NA	1	0

Smith et al. [18] (2016)	1	26	M	7 days	Jaundice, fatigue	Hepatocellular	NA	1	0

Lunsford et al. [19] (2016)	1	34	M	150 days	Nausea, vomiting, abdominal pain, and dark urine	Hepatocellular	NA	1	0

Kothadia et al. (present case)	1	26	F	28 days	Fever, nausea, vomiting, abdominal pain, malaise, fatigue, and jaundice	Hepatocellular	8	0	0

^*∗*^Mean age; M: male; F: female; GC: *Garcinia cambogia*; ^*¥*^mean score; NA: not applicable.
